# Exploring the limits of scan time reduction for ferumoxytol-enhanced whole-heart angiography in congenital heart disease patients

**DOI:** 10.1016/j.jocmr.2025.101854

**Published:** 2025-02-05

**Authors:** Ludovica Romanin, Milan Prsa, Christopher W. Roy, Xavier Sieber, Jérôme Yerly, Bastien Milani, Tobias Rutz, Salim Si-mohamed, Estelle Tenisch, Davide Piccini, Matthias Stuber

**Affiliations:** a Department of Radiology, Lausanne University Hospital and University of Lausanne, Lausanne, Switzerland; b Advanced Clinical Imaging Technology, Siemens Healthineers International AG, Lausanne, Switzerland; c Division of Pediatric Cardiology, Woman-Mother-Child Department, Lausanne University Hospital and University of Lausanne, Lausanne, Switzerland; d Center for Biomedical Imaging (CIBM), Lausanne, Switzerland; e Service of Cardiology, Lausanne University Hospital and University of Lausanne, Lausanne, Switzerland; f University Lyon, INSA-Lyon, University Claude Bernard Lyon 1, UJM-Saint Etienne, CNRS, Inserm, CREATIS, Villeurbanne, France; g Department of Radiology, Louis Pradel Hospital, Bron, France

**Keywords:** Ferumoxytol, Whole-heart, Cardiac MRI, CMR, 3D angiography, Pediatric MR

## Abstract

**Background:**

One major challenge in cardiovascular magnetic resonance is reducing scan times to be more compatible with clinical workflows. In 3D magnetic resonance imaging (MRI), strategies to shorten scan times mostly rely on ECG‐triggering or self-navigation for motion management, but are affected by heart rate variabilities or respiratory drifts. A similarity-driven multi-dimensional binning algorithm (SIMBA) was introduced for 3D whole-heart angiography from ferumoxytol-enhanced free-running MRI. This study explores acceleration limits using SIMBA, and its compressed-sensing extension extra-dimensional motion-compensation (XD-MC)-SIMBA, while preserving image quality.

**Methods:**

Data from 6-min free-running acquisitions of 30 congenital heart disease (CHD) patients were retrospectively undersampled to simulate 5-, 4-, 3-, 2-, and 1-min datasets. SIMBA and XD-MC-SIMBA reconstructions were applied. and the consistency of the data selection together with sharpness metrics were computed as a function of undersampling. Image quality was rated on a 5-point Likert scale. Shorter 3-minute acquisitions were prospectively acquired in nine CHD patients.

**Results:**

SIMBA's motion state selection was consistent across undersampling levels, with only 2 of 30 cases showing completely different selections. Image quality metrics decreased with increased undersampling, with SIMBA scoring lower compared to XD-MC-SIMBA. The diagnostic quality was good, with lower scores for 2- and 1-min datasets. Using XD-MC-SIMBA, 43% (31/72) of cases showed improved scores compared to SIMBA and 58% (7/12) of 1-min datasets improved to good or excellent quality.

**Conclusions:**

This study demonstrates that ferumoxytol-enhanced free-running MRI can be highly accelerated for 3D angiography in CHD.With the aid of compressed sensing, XD-MC-SIMBA supports the acceleration down to 3 minutes or less.

## Graphical abstract

**Figure:**
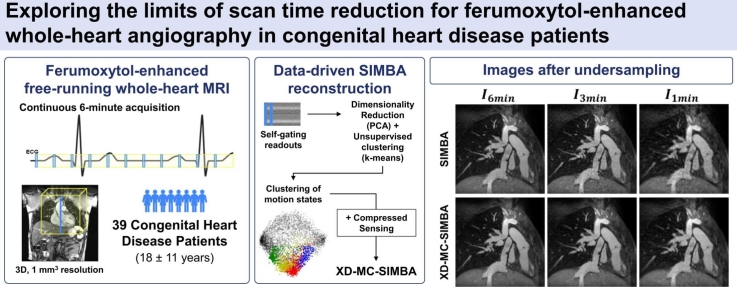

## 1 Introduction

Magnetic resonance angiography (MRA) is becoming a powerful tool for evaluating cardiac anatomy and vasculature in patients with coronary artery disease [1] or congenital heart disease (CHD) [2], due to its radiation-free nature and high soft tissue contrast. However, its clinical uptake is hindered by prolonged scan times. Therefore, accelerating magnetic resonance imaging (MRI) acquisition is a major research focus [3], [4], [5]. Advances in image reconstruction methods for parallel imaging [6], [7], [8] and compressed sensing [9], [10], [11] have enabled image generation from highly undersampled k-space data. These techniques exploit redundancies in the spatial, temporal, or contrast dimensions to reduce the amount of acquired data needed and therefore support abbreviated scan times. In three-dimensional (3D) MRA, several strategies have been proposed to shorten scan times by exploiting temporal redundancies as in extra-dimensional golden-angle radial sparse parallel (XD-GRASP) [12], [13], or spatial redundancies as in LOw-dimensional-structure Self-learning and Thresholding (LOST)[14] or 3D-patch-based low-rank reconstruction (PROST) [3]. However, these approaches rely on motion management methods (e.g. ECG‐triggering or self-navigation) and are susceptible to heart rate variabilities or respiratory drifts, resulting in longer and unpredictable scan times. Non-triggered and ungated techniques, such as free-running MRI [15], [16], [17], have been proposed to simplify acquisition planning with isotropic spatial resolution and allow for flexible and data-driven reconstruction strategies. In this context, a similarity-driven multi-dimensional binning algorithm (SIMBA) [18] was introduced to reconstruct free-running whole-heart MRI data without explicitly predefining physiological phases, by combining similar motion states occurring over a 6-minute gradient recalled echo (GRE) acquisition. Coupled with contrast enhancement using the ultrasmall superparamagnetic iron oxide ferumoxytol, SIMBA allows for an angiographic evaluation of cardiac anatomy [18], [19].

The use of ferumoxytol has shown very promising results in pediatric cardiac MRI, particularly for imaging CHD, enabling sub-millimetric isotropic whole-heart coverage even in very small infants [2]. Han et al. demonstrated the benefits of ferumoxytol-enhanced angiography with ECG and ventilator-gating (MUSIC [20]) or with self-gating (ROCK-MUSIC [21]). However, both techniques involved data acquisition under general anesthesia and positive pressure ventilation. While general anesthesia and intubation are commonly used for easier motion management in difficult and uncooperative pediatric patients, this requires additional overhead in personnel and cost. Roy et al. proposed a fully automated motion-resolved ungated and free-breathing angiography technique, resulting in non-significant differences in image quality between sedated and non-sedated CHD patient cohorts [22]. Although the scan duration in this report was 5 min and 59 s to obtain cardiac and respiratory-resolved 5D images, the boundaries of scan time reduction for static 3D whole-heart angiography with SIMBA reconstruction, to improve workflow as part of a clinical protocol, have never been investigated for pediatric imaging.

The goal of this work was to evaluate whether the data-driven SIMBA reconstruction and its compressed-sensing extension XD-MC-SIMBA could support the acceleration of ferumoxytol-enhanced free-running MRI acquisitions. Specifically, we leveraged the higher blood-pool signal provided by ferumoxytol to assess the feasibility of shorter scan times for 3D whole-heart MRA. Performing time-efficient and easy-to-use free-breathing 3D whole-heart MRI in non-sedated pediatric CHD patients can have a considerable impact on patient management and promote a more widespread use of MRA in such patient cohorts.

## 2 Methods

### 2.1 Study of ferumoxytol-enhanced free‑running MRI acquisitions acceleration

Analysis of de-identified data not including health-related personal data does not constitute human subjects research in accordance with the Human Research Ordinance (Art. 6, HRO), on the basis of the Swiss Human Research Act.

In vivo data were acquired on a 1.5T clinical MRI scanner (MAGNETOM Sola, Siemens Healthineers, Erlangen, Germany). A GRE-based free-running custom research sequence without pre-pulses was applied as described by Roy et al. [22]. A 3D golden-angle koosh-ball phyllotaxis trajectory [23] was used to continuously acquire k-space data, interleaved with the sampling of superior-inferior (SI) readouts, used for motion correction, at a frequency of 16 Hz. Main sequence parameters were radio frequency excitation angle of 15° with an axial slab-selective sinc pulse, resolution of 1.15 mm^3^, field-of-view (FOV) of 220 mm^3^, TE/TR of 1.64/2.84 ms, and a readout bandwidth of 1002 Hz/pixel. The slab-selective pulse is of size equal to the prescribed FOV, and it is used to minimize unwanted signal from the head, shoulders, and lower abdomen. All exams were performed during free-breathing, after administration of 1–5 mg/kg of ferumoxytol (Feraheme, AMAG Pharmaceuticals, Waltham, Massachusetts, USA), infused over 15 min. Blood pressure was routinely measured before, immediately after, and 30 min after injection of ferumoxytol. None of the patients had significant changes in their blood pressure. There were no patients who had adverse events during or after ferumoxytol administration. The ECG signal was recorded as a reference.

Data were collected from a cohort of 30 consecutively recruited CHD patients. Table 1 summarizes CHD diagnoses and clinical indications for all patients. The patient population consisted of 20 males and 10 females, with ages ranging from 7 months to 53 years (mean age: 19.6 years ± 11.8 years) and weight ranging from 5–104 kg (mean weight: 57.6 kg ± 24.3 kg). The acquisition protocol consisted of 5749 radial interleaves and 22 readouts/interleave, corresponding to an undersampling factor of approximately 0.5. We note that the total amount of acquired data with a free-running sequence leads to an oversampled dataset to ensure, in the presence of motion, enough information per phase after the binning. The 30 datasets were retrospectively undersampled along the interleaves dimension, by sequentially removing from the full acquisition ($I_{6\min}$) the last 1 min of data for $I_{5\min}$, the last 2 min for $I_{4\min}$, the last 3 min for $I_{3\min}$, the last 4 min for $I_{2\min}$, and the last 5 min for $I_{1\min}$. This ensures that the temporal order in which the data were acquired was maintained for all datasets. This corresponds to undersampling factors of 0.6 for $I_{5\min}$, 0.7 for $I_{4\min}$, 0.9 for $I_{3\min}$, 1.4 for $I_{2\min}$, and 2.9 for $I_{1\min}$. These datasets were then reconstructed into $I_{6\min}$, $I_{5\min}$, $I_{4\min}$, $I_{3\min}$, $I_{2\min}$, and $I_{1\min}$ images, corresponding to the full acquisition, the first 5, 4, 3, 2, and 1 min of data, respectively. Image reconstruction was performed with both SIMBA [18] and XD-MC-SIMBA [19] techniques.

**Table 1:** Summary of information, congenital heart disease (CHD) diagnoses, and clinical indications for all patients in the retrospective study.

*General information*	*Mean*	*SD*	*Count^a^*
Age	19	12	
Weight	57	24	
Sex: male			20
Sex: female			10
		
*CHD diagnoses*	*Clinical indications*		
D-transposition of the great arteries	Branch pulmonary artery stenosis		6
Fontan	Fontan surveillance		5
Coarctation of the aorta	Recoarctation		3
Pulmonary atresia with ventricular septal defect	Right ventricular outflow tract obstruction; pulmonary insufficiency		3
Tetralogy of Fallot	Pulmonary insufficiency		2
Connective tissue disease	Dilated aortic root		1
Total anomalous pulmonary venous connection	Pulmonary vein stenosis		1
Common arterial trunk	Branch pulmonary artery stenosis		1
Status-post Ross procedure	Right ventricular outflow tract obstruction		1
Marfan syndrome	Dilated aortic root		1
Interrupted aortic arch with isolated left pulmonary artery	Branch pulmonary artery stenosis		1
Sinus venosus atrial septal defect with partial anomalous pulmonary venous connection	Pre-operative assessment		1
Double aortic arch	Pre-operative assessment		1
Isolated hypoplasia of the right ventricle	Evaluation of right ventricular volume and function		1
Mitral valve prolapse	Mitral insufficiency		1
Ventricular septal defect with double-chambered right ventricle	Pre-operative assessment		1

*SD standard deviation*

^a^Data are number of cases.

### 2.2 Similarity-driven reconstructions for free-running 3D angiography

**Fig. 1:**
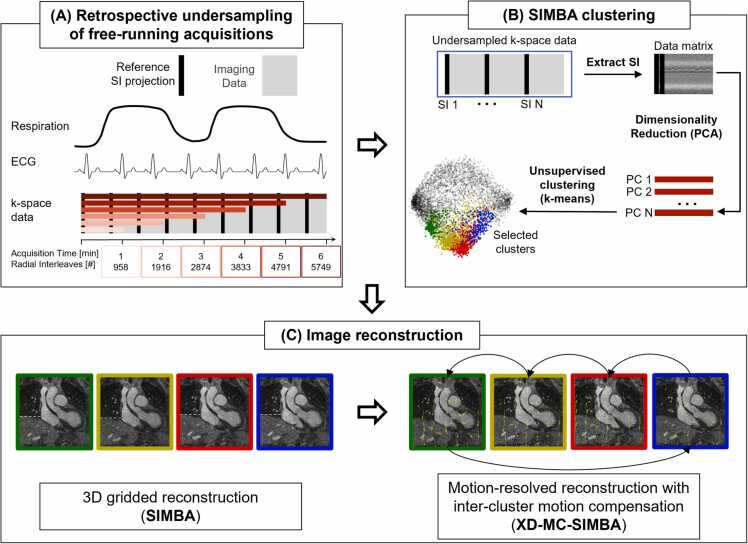
Acquisition and reconstruction pipeline. From a free-running acquisition, the k-space data are subdivided into six datasets of different durations, which are obtained by progressively removing 1 min of data from the end of the acquisition to obtain a 5-, 4-, 3-, 2-, and 1-min datasets (A). To each of these datasets, the SIMBA clustering was applied. The algorithm takes as input the reference SI readouts concatenated into a matrix, which is then reduced in dimension using principal component analysis (PCA). These components are then clustered into a set of disjoint groups, each corresponding to a different motion state (B). (C) A subset of these clusters is reconstructed using a non-uniform 3D gridded reconstruction and for analysis only the one resulting from the largest cluster is considered (SIMBA). A motion-resolved reconstruction with inter-cluster motion compensation is obtained by performing a compressed-sensing reconstruction with regularization over the clustering dimension (XD-MC-SIMBA). *SIMBA* similarity-driven multi-dimensional binning algorithm, *ECG* electrocardiogram, *3D* three-dimensional, *PC* principal component, *SI* superior-inferior

All data were reconstructed with a SIMBA, as published by Heerfordt et al. [18]. This technique performs a very fast binning (below 20 s [18]) of the data in a purely data-driven way, as a combination of principal component analysis for dimensionality reduction and feature extraction, and k-means for unsupervised clustering of these reduced data into 7–14 disjoint clusters, according to similarity. Each cluster was reconstructed with a 3D gridded reconstruction for non-Cartesian acquisitions, consisting of Voronoi density compensation [24], [25], non-uniform fast Fourier transform (NUFFT) [26], and coil sensitivities estimated from the pre-scan calibration data [6], [25]. For diagnosis, only the image from the largest cluster was considered. To further improve image quality, data were also reconstructed with a compressed-sensing motion-resolved reconstruction along the SIMBA cluster dimension (XD-MC-SIMBA) [19]. A summary of the reconstruction pipeline is shown in Fig. 1. Reconstructions were performed offline and the computation times were recorded.

### 2.3 Analysis of the data selection

To evaluate the robustness of SIMBA clustering and data selection during retrospective sequence accelerations, the number of selected interleaves was measured. Their uniformity in k-space was analyzed by computing their distribution on a unit sphere as the relative standard deviation of the distance between interleaves and their four closest neighbors (SD%). This metric reflects how sparsely the data are distributed in k-space, with lower values indicating a more uniform coverage [18]. Moreover, we ascertained the percentage of data overlap between the readouts selected for each undersampling level ($D_{I_{x\min}}$, with x=5,4,3,2,1min) and those selected in the full acquisition $D_{I_{6\;\min}}$ as

$\%\;\text{data overlap}=\;\frac{D_{I_{x\min}}\cap \;D_{I_{6\;\min}}}{\text{size}(D_{I_{x\min}})}x\;100$

for x=5,4,3,2,1min. We divide by $\text{size}(D_{I_{x\min}})$ to remove the effect of the size of the cluster. This value is at 100% if there is a perfect selection of the same motion state after SIMBA clustering, while 0% indicates that two completely distinct motion states have been selected.

### 2.4 Image quality analysis

To compare changes in image quality as a function of undersampling, we measured the degree of focus by computing the discrete cosine transform energy ratio (DCTE), a focus measure equal to the mean of ratios between high-frequency components and the mean intensity, across the whole image [27]. The advantage of using the DCTE is that it is unbiased by the presence of noise and it performs equally well in low and high contrast images [28]. The DCTE was computed on each slice separately, after cropping the images around the heart, and then averaged to get one value per volume. Higher DCTE corresponds to a higher degree of focus in the image, while a lower DCTE to blurrier images. Additionally, degradation in image quality was assessed with the structured similarity index (SSIM) [29], which considers the perceived change in structural information and is equal to 1 for perfect similarity. The SSIM was calculated on the whole 3D volumes between $I_{6\min}$ and all the other images ($I_{5\min}$, $I_{4\min}$, $I_{3\min}$, $I_{2\min}$, $I_{1\min}$). In addition to these automated quantitative metrics, the sharpness of the aorta was measured by manually selecting a slice at the insertion point of the right coronary artery, for maximized reproducibility and precision in the measurement. Moreover, we computed also the average diameter and sharpness of the proximal 4 cm of the left anterior descending (LAD) coronary artery, after manual segmentation.

### 2.5 Diagnostic quality evaluation

A diagnostic quality 5-point-scale Likert score was assigned independently to each image by a cardiologist with 12 years of experience (M.P.) and by a radiologist with 12 years of experience (E.T.), blinded to the type of image. The scale used was the following: non-diagnostic quality (0: impossible to answer the clinical questions), limited quality (1: difficult but still possible to answer the clinical questions), acceptable quality (2: adequate for answering the clinical questions), good quality (3: easy to answer the clinical questions), and excellent quality (4: extremely easy to answer the clinical questions). Images were shown in a random, blind order, for both undersampling levels and patients. For the retrospective analysis, only a subset of 12 randomly selected datasets was shown. This was done for both SIMBA and XD-MC-SIMBA reconstructed images. The count of visible coronary ostia for both right and left coronary arteries was ascertained visually by L.R. (4 years of experience in cardiovascular magnetic resonance).

### 2.6 Prospective scans

To evaluate the effects of scan time acceleration prospectively, a smaller cohort of 9 CHD patients (Table 2) was scanned with a protocol that included the same 22 readouts/interleave but only 2932 shots, taking 3:03 min. To determine this accelerated scan duration, we analyzed the results from the retrospective study using the image quality metrics, together with the visibility of anatomical structures relevant to the clinical indication (coronary ostia visibility and image quality scores). The 3-min datasets showed no statistical difference in terms of metrics compared to the original 6-min datasets and maintained the same visibility of anatomical structures, in both SIMBA and XD-MC-SIMBA. The same reconstruction pipeline as that used for the retrospective study was applied to these acquisitions. As for the retrospective data analysis, the SIMBA data selection was characterized in terms of uniformity in k-space (SD%). The average DCTE was also computed. Quality scores were assigned independently by the same two readers and with the Likert scale identical to that used for the retrospective analysis.

**Table 2:** Summary of information, congenital heart disease (CHD) diagnoses, and clinical indications for the patients included in the prospective study.

*General information*	*Mean*	*SD*	*Count^a^*
Age	11	2	
Weight	40	22	
Sex: male			7
Sex: female			2
		
*CHD diagnoses*	*Clinical indications*		
Tetralogy of Fallot	Pulmonary insufficiency; pre-operative assessment; branch pulmonary artery stenosis		4
D-transposition of the great arteries	Branch pulmonary artery stenosis		1
Coarctation of the aorta	Recoarctation		1
Connective tissue disease	Dilated ascending aorta		1
Bicuspid aortic valve	Dilated ascending aorta		1
Common arterial trunk	Aortic insufficiency		1

*SD standard deviation*

^a^Data are number of cases.

### 2.7 Statistical analysis

Data were compared using the Paired Samples t-test, with p < 0.01 considered statistically significant, after Bonferroni correction for multiple comparisons.

## 3 Results

### 3.1 Retrospective analysis

**Fig. 2:**
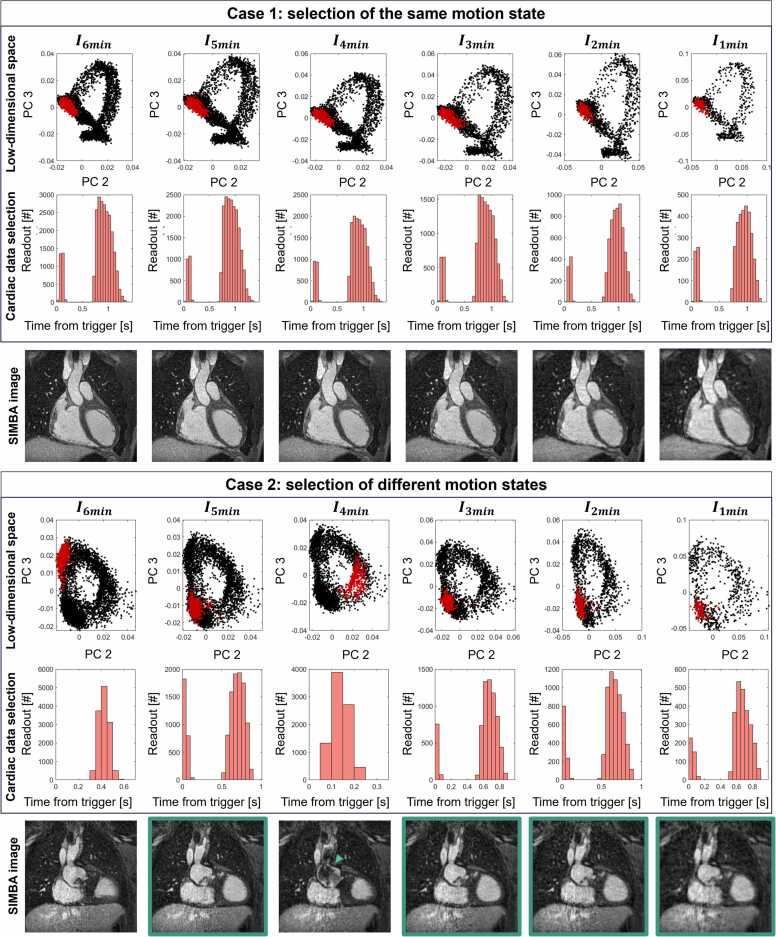
Example cases showing the data selection using SIMBA for all undersampling levels. For each case, highlighted in red are the readouts in the largest cluster selected using SIMBA. The low-dimensional space corresponds to the projection of the SI readouts in the principal component space. The cardiac data selection consists of plotting these selected readouts in terms of their acquisition time from the R-wave (trigger) time, obtained from the ECG signals. Case 1: the data selected using SIMBA are in the same motion state for all undersampling levels, as it can be clearly seen by having the same location of the low-dimensional space selected. This is mirrored by having the same cardiac phase selected, which according to the time distribution, corresponds to a diastolic phase of the heart. The reconstructed images are all depicting the same anatomy. Patient with a total anomalous pulmonary venous return, age 19, and weight 66 kg. The ferumoxytol dose was 2 mg/kg. Case 2: the data selected using SIMBA change for the different undersampling levels, as it can be visualized in the different locations of the low-dimensional space that are reconstructed. For $I_{6\min}$, the image reconstructed is in a mid-systolic phase, while for $I_{5\min}$, $I_{3\min}$, $I_{2\min}$, and $I_{1\min}$ it is in a diastolic phase of the heart. $I_{4\min}$ represents a systolic phase of the heart, also made clear by the strong flow-dephasing artifacts in the aorta (arrow). Patient with a common arterial trunk, age 14, and weight 47 kg. The ferumoxytol dose was 3 mg/kg. *SIMBA* similarity-driven multi-dimensional binning algorithm, *SI* superior-inferior, *ECG* electrocardiogram, *PC* principal component

**Fig. 3:**
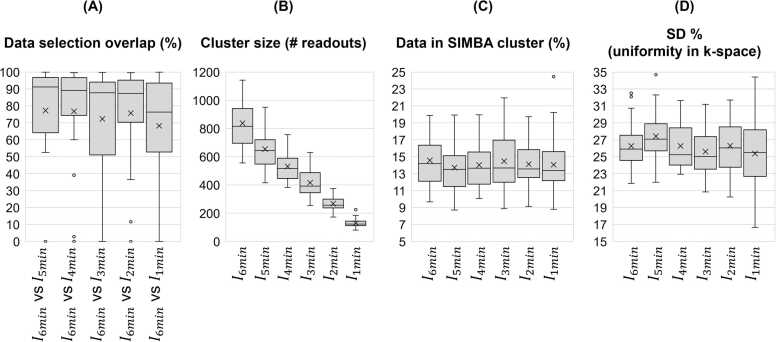
Data selection metrics. (A) Percentage overlap between the data selected in the 6-min acquisition, and those from the other datasets. It can be seen how there are a few cases of no overlap (0%), and how the overlap decreases with increased undersampling, with lowest percentages for the 1-min datasets. (B) The size of the cluster for all undersampling levels as the number of readouts selected per interleave, in the largest cluster reconstructed. (C) The percentage of the data reconstructed (SIMBA cluster) with respect to the total amount of data acquired, after the retrospective undersampling. (D) The percentage standard deviation of the selected data in k-space, as a measure of the uniformity in k-space. *SIMBA* similarity-driven multi-dimensional binning algorithm, *SD* standard deviation

Qualitatively, the motion states selected by the SIMBA clustering are the same across all levels of undersampling, with a consistent selection of the same cardiac phase (Fig. 2, Case 1), as confirmed by using the recorded ECG as a reference. In only 2 out of 30 cases, the state in $I_{6\min}$ was different from all other states in $I_{5\min}$, $I_{4\min}$, $I_{3\min}$, $I_{2\min}$, and $I_{1\min}$ (Fig. 2, Case 2). In five additional cases, we observe the selection of different states across all undersampling levels. The percentage of data overlap was in a similar range for all undersampling levels, and it was zero in the case of no overlap (Fig. 3A).

The number of selected interleaves using SIMBA, or the cluster size, decreased with an increased level of undersampling (Fig. 3B), but the percentage of data selected with respect to the total amount of data acquired was overall the same for all undersampling levels (Fig. 3C), in line with the trend observed for the percentage of data overlap. The uniformity in k-space, SD%, was also the same on average for all cases (Fig. 3D). Compared to the full acquisition, there was no statistical difference for the means of the percentage of data selected (p > 0.12) and no statistical difference for the means of SD% (p > 0.18).

**Fig. 4:**
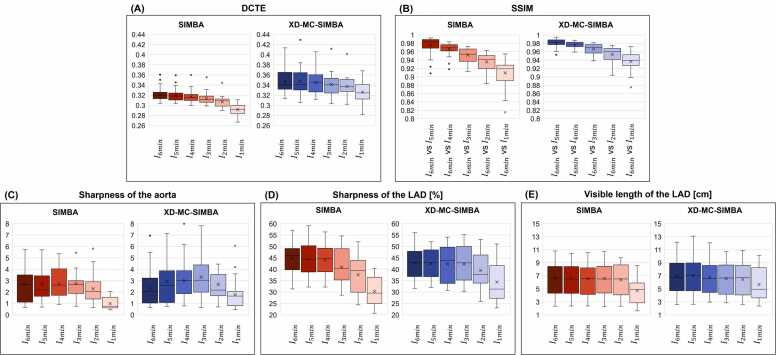
Image quality metrics. (A) The discrete cosine transform energy ratio (DCTE) focus measure computed for each undersampling level, for both SIMBA and XD-MC-SIMBA reconstructions. For both reconstruction types, values decrease with increased undersampling, but at a lower rate for XD-MC-SIMBA and values are overall higher. (B) The structured similarity index (SSIM) computed between the image from the 6-min scan ($I_{6\min}$) and $I_{5\min,\ldots ,1\min}$. (C) The sharpness of the aorta was computed. (D) The sharpness of the left anterior descending coronary artery (LAD). (E) The visible length of the LAD was traced on reformatted images. *SIMBA* similarity-driven multi-dimensional binning algorithm

The focus measure DCTE decreased with undersampling, with lower values in SIMBA reconstructions compared to XD-MC-SIMBA (Fig. 4A). With SIMBA, for $I_{5\min}$, $I_{4\min}$, and $I_{3\min}$, the mean DCTE was statistically not different compared to that of $I_{6\min}$ (p = 0.63 for $I_{5\min}$, p = 0.20 for $I_{4\min}$, and p = 0.019 for $I_{3\min}$). With XD-MC-SIMBA, this trend was observed even down to $I_{2\min}$ (p = 0.98 for $I_{5\min}$, p = 0.61 for $I_{4\min}$, p = 0.30 for $I_{3\min}$, and p = 0.08 for $I_{2\min}$).

Compared to $I_{6\min}$, the SSIM decreased from $I_{5\min}$ to $I_{1\min}$ with SIMBA, but at a reduced rate with XD-MC-SIMBA (Fig. 4B). With SIMBA, the sharpness of the aorta (Fig. 4C) decrease is almost the same from $I_{6\min}$ to $I_{3\min}$ (variations of ±0.03; p > 0.9) and goes down for $I_{2\min}$ (p = 0.29) and significantly for $I_{1\min}$ (p < 0.0001). With XD-MC-SIMBA, the sharpness of the aorta is overall higher for all undersampling levels compared to SIMBA, with no level being statistically different compared to $I_{6\min}$ (p > 0.05). Similar observations can be made for the sharpness of the LAD (Fig. 4D). For both SIMBA and XD-MC-SIMBA, the visible length of the LAD (Fig. 4E) stays invariant from $I_{6\min}$ down to $I_{2\min}$, with lower values for $I_{1\min}$ but not statistically significant (p > 0.05).

**Fig. 5:**
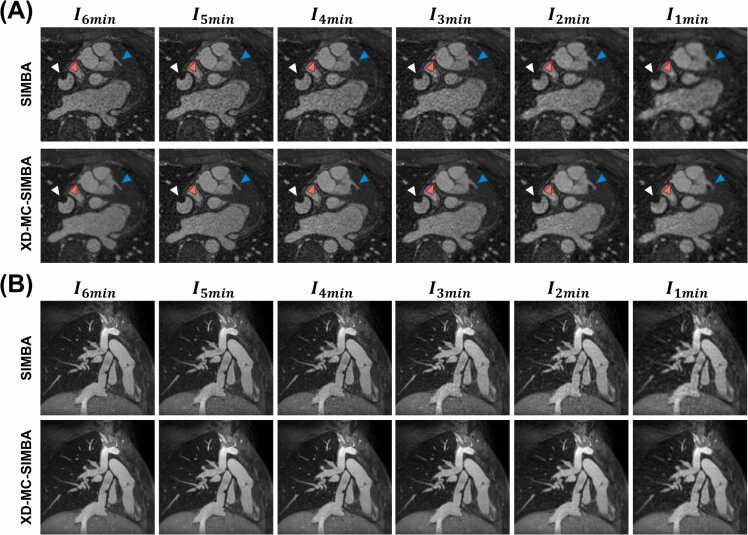
Example of patient with a Fontan circulation (age: 20 years; weight: 68 kg, ferumoxytol dose: 2 mg/kg) showing the effects of undersampling on image quality. (A) The data were reconstructed with both SIMBA and XD-MC-SIMBA. For 6-, 5-, 4-, and 3-min datasets, the images do not show significant differences in the anatomical structures or vessel delineation (red arrow indicates the right coronary ostium, blue arrow indicates the left coronary ostium). There is a decrease in image quality for SIMBA for the 2- and 1-min datasets, which is improved when applying XD-MC-SIMBA. The white arrow indicates an artifact coming from a device that was used to close the fenestration in the Fontan conduit. (B) For the same patient, reformat to show the total cavopulmonary connection, clearly visible for all undersampling levels. *SIMBA* similarity-driven multi-dimensional binning algorithm

**Fig. 6:**
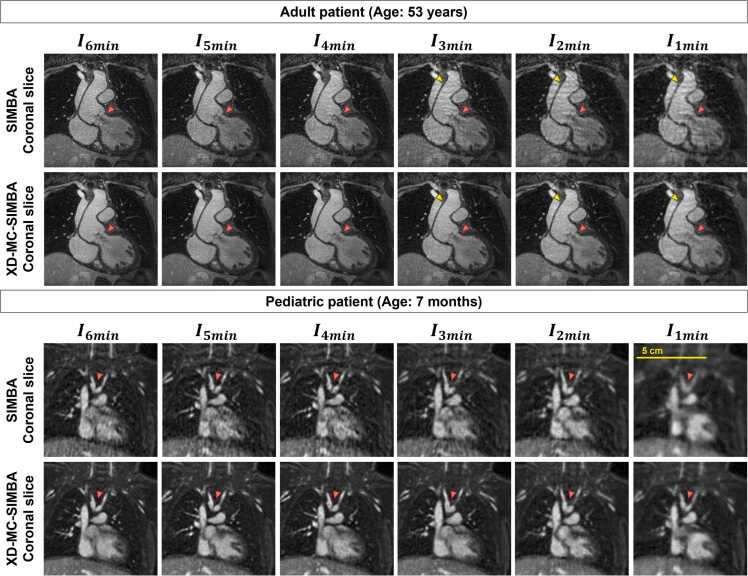
Example of image quality for the oldest adult patient (age: 53 years, weight: 65 kg, ferumoxytol dose: 2 mg/kg) and the youngest pediatric patient (age: 7 months, weight: 5.1 kg, ferumoxytol dose: 5 mg/kg) in our cohort. For the adult case, there is a visible increase in streaking artifacts with higher undersampling factors, which are highly attenuated with XD-MC-SIMBA. For the pediatric patient, the undersampling causes additional blurring, which decreases with XD-MC-SIMBA. *SIMBA* similarity-driven multi-dimensional binning algorithm

Qualitatively, images $I_{6\min}$ to $I_{4\min}$ do not show visible differences, even for higher undersampling levels. Although blurrier, all major anatomical structures remain visible (Figs. 5 and 6). For all undersampling levels, the right coronary ostia were always visible in 10 out of 30 patients for SIMBA, and always visible in 23 cases out of 30 for XD-MC-SIMBA. Similarly, the number of visible left coronary ostia increased for XD-MC-SIMBA (19/30 cases) compared to SIMBA (12/30 cases). In three patients, the right coronary was not visible, while the left coronary was not visible in eight patients (Table 3).

**Table 3:** Count of visible ostia for both right and left coronary arteries for n = 30 patients.

	Reconstruction algorithm											
Metrics	SIMBA						XD-MC-SIMBA					
Undersampling levels	6 min	5 min	4 min	3 min	2 min	1 min	6 min	5 min	4 min	3 min	2 min	1 min
Visible RCA ostium	27	26	26	26	23	10	27	26	26	26	23	23
Visible LM ostium	22	22	21	22	18	12	22	22	21	22	20	19

For the RCA, the number of visible ostia is the same for both SIMBA and XD-MC-SIMBA for the 6-, 5-, 4-, 3-, and 2-min datasets, while for the 1-min datasets, XD-MC-SIMBA greatly improves the visibility. For three patients, the RCA was not visible. For the LM, SIMBA and XD-MC-SIMBA provide equal visibility of the ostia for the 6-, 5-, 4-, and 3-min datasets, while for the 2- and 1-min datasets, XD-MC-SIMBA increases the visibility. For eight patients, the LM was not visible. Data are number of cases.

*SIMBA* similarity-driven multi-dimensional binning algorithm, *RCA* right coronary artery, *LM* left main coronary artery

Reconstruction times for XD-MC-SIMBA decreased with acceleration: 39 min ± 3 min for $I_{6\min}$, 36 min ± 1 min for $I_{5\min}$, 34 min ± 1 min for $I_{4\min}$, 33 min ± 2 min for $I_{3\min}$, 30 min ± 1 min for $I_{2\min}$, and 30 min ± 2 min for $I_{1\min}$. These times are approximately 40–30 times higher than those of SIMBA (1 min ± 25 s).

### 3.2 Diagnostic quality evaluation

**Fig. 7:**
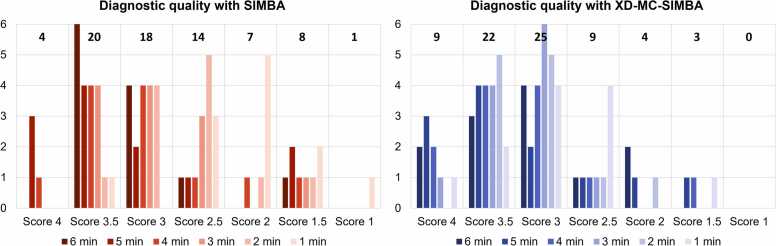
Diagnostic image quality scores using a 5-point Likert scale. Twelve randomly selected patients were blindly graded with scores of 0 = non-diagnostic image quality, 1 = limited diagnostic quality, 2 = acceptable diagnostic quality with artifacts, 3 = good diagnostic quality, and 4 = excellent diagnostic quality. In the bar plots are reported the counts of images assigned per quality score level, labeling the undersampling level. Only three cases had non-diagnostic quality in XD-MC-SIMBA, while more cases (nine) were scored as non-diagnostic in SIMBA. *SIMBA* similarity-driven multi-dimensional binning algorithm

**Fig. 8:**
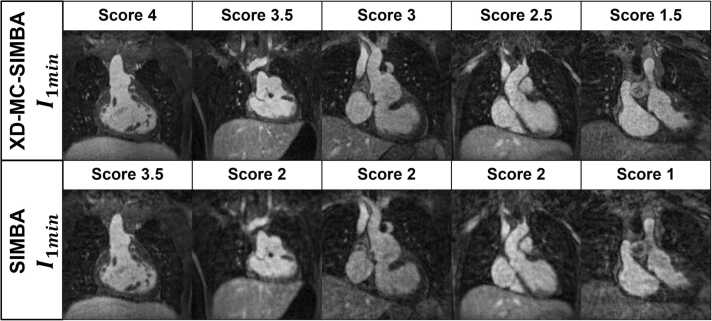
Example images from the 1-min datasets for each Likert score. Ferumoxytol doses (in mg/kg) for each patient (left to right) were 2, 4, 2, 3.7, 2. *SIMBA* similarity-driven multi-dimensional binning algorithm

On average, the diagnostic quality scores were good, with limited or acceptable diagnostic values mostly assigned to $I_{2\min}$ and $I_{1\min}$ (Fig. 7). Only in 1 out of the 30 cases were the scores below good quality for all undersampling levels (in Supplementary Information, Fig. S1). XD-MC-SIMBA resulted in higher diagnostic values for 43% (31/72) of cases and allowed to reach above acceptable (>2.5) to excellent image quality in 58% (7/12) of $I_{1\min}$ cases. Images with lower quality scores are blurrier, with small structures such as the coronary arteries hard or even impossible to see (Fig. 8).

### 3.3 Prospective scans

**Fig. 9:**
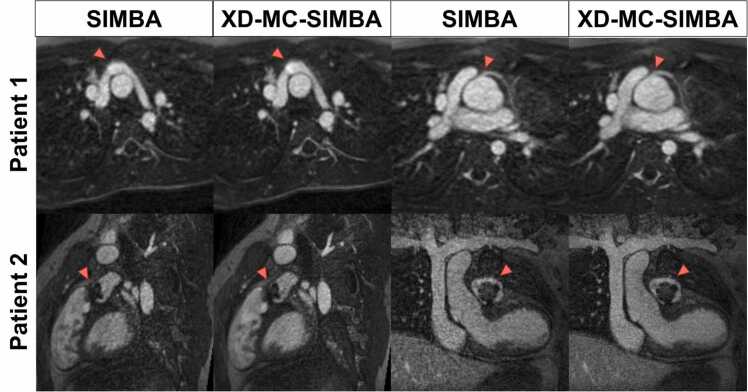
Example patients from the prospectively shortened acquisitions of 3 min. Patient 1 is an 11-year-old with status-post arterial switch operation for D-transposition of the great arteries (weight: 32 kg, ferumoxytol dose: 3 mg/kg). The images on the left show the pulmonary arteries, while the images on the left the reimplanted left coronary artery. Patient 2 is a 17-year-old patient with repaired tetralogy of Fallot (weight: 100 kg, ferumoxytol dose: 2 mg/kg). In patient 2, we see an artifact from a percutaneously implanted pulmonary valve, mounted on a stent (red arrows). All images were graded as of good diagnostic quality to answer the clinical question. XD-MC-SIMBA only slightly improves the global image quality but helps in achieving increased conspicuity of smaller vessels such as the coronary arteries. *SIMBA* similarity-driven multi-dimensional binning algorithm

Images were successfully reconstructed for all nine acquired patients, with cardiac anatomy and vessels clearly visible with both SIMBA and XD-MC-SIMBA reconstructions (Fig. 9). XD-MC-SIMBA slightly improved image quality compared to SIMBA, with a reduction in noise and better discrimination of structures. The average DCTE was 0.24 ± 0.03 for SIMBA and 0.25 ± 0.03 for XD-MC-SIMBA, which is slightly lower compared to 0.31 ± 0.01 SIMBA and 0.34 ± 0.02 XD-MC-SIMBA for $I_{3\min}$ in the retrospective study. The uniformity in k-space was on average higher for these scans compared to the retrospectively accelerated data (26.15 ± 2.7 vs 25.61 ± 2.7). All cases were scored above 2.5 for SIMBA (3 ± 0.4) and above 3 for XD-MC-SIMBA (3.2 ± 0.3).

## 4 Discussion

This study demonstrated the performance of the data-driven reconstruction SIMBA and its extension XD-MC-SIMBA in progressively accelerated ferumoxytol-enhanced free-running MRI acquisitions. The results show that data selection using SIMBA clustering is consistent and robust for all undersampling levels, and resultant reconstructed images maintain high and almost identical image quality down to 3-min durations. With XD-MC-SIMBA, even 2- and 1-min datasets provide high-quality diagnostic images. The 3-min scans resulted in images of good diagnostic quality, for both SIMBA and XD-MC-SIMBA.

Our results highlight the strength of the SIMBA technique in robustly producing motion-free volumes of the heart, even for highly accelerated acquisitions. Thanks to the ECG recorded as a reference, we could assess the consistency in selecting the same cardiac phase. In only two cases, the data selected by SIMBA in the shortened acquisitions did not overlap with that selected in the full 6-min acquisition. In five additional cases, there was the selection of different states across undersampling levels. This comes from the nature of the data-driven SIMBA clustering, as by selecting the largest cluster we assume to always pick the same resting phase of the heart but do not have any control over the data selected. However, these images are of equally high diagnostic quality. These swaps in the selected state could also be an indication that something happened in that portion of the scan, such as a sudden heart rate increase or the presence of bulk motion.

SIMBA clustering was applied with identical parameters for all datasets, ensuring consistent clustering of motion states even for very short scans. Consequently, the percentage of data selected compared to the total acquired data remained almost the same for all undersampling levels. The data uniformity in k-space (SD %) also remained consistent even for very highly undersampled and sparser datasets of only 1 min. Even for short acquisitions capturing a few cardiac cycles, SIMBA should cluster the different states by similarity. However, this study did not evaluate the performance of SIMBA clustering in terms of the number of heartbeats captured in the acquisition, which could be an additional analysis to consider when defining the limit of scan time reduction.

This retrospective study used a large dataset, including 360 images in total. To ensure an unbiased comparison of images for different undersampling levels and reconstruction techniques, we employed automated methods to calculate image quality metrics. Both DCTE and SSIM provided insights into the impact of undersampling levels on final image quality, without requiring manual segmentations. However, these metrics alone do not entirely reflect the diagnostic quality of the images, so we included Likert scoring from a pediatric cardiologist to corroborate our findings.

The patient cohort considered in this study received different doses of ferumoxytol, depending on age and weight. Usually, adult patients (>18 years) do not receive doses above 2 mg/kg. Lower doses (3 mg/kg or below) are also administered to larger patients (≥60 kg). It is important to note that the use of ferumoxytol for imaging is still an off-label use of commercially available ferumoxytol to treat anemia. Consequently, vials are typically of 510 mg per 17 mL or 30 mg/mL. Although there is a meticulous planning to have patients of complementary weights consecutively in the same day, this is not always possible. To avoid being wasteful, some patients receive doses slightly higher or lower depending on what is left in the vial. Guidelines report doses in the range of 1–5 mg/kg for imaging purposes [30], and recent studies show that above 3 mg/kg, there is no significant increase (or differences) in CNR and SNR. Moreover, in the same study, it is mentioned how for larger patients lower doses could lead to the same optimal results [31]. Consequently, we did not take into account the dose in our analysis as the non-linear relaxation behavior of ferumoxytol in blood [32] would require subdividing the patients based on their intravascular blood volume. In this study, we are addressing the reduction in undersampling artifacts with our published reconstruction techniques. By testing this highly heterogeneous patient population (different ages and sizes), we compensate possible signal differences and make our findings generalizable.

## 5 Limitations

This study compares image quality from a set of acquisitions retrospectively shortened by progressively removing minutes of data from the end. Redistributing data in different motion states, regardless of the moment the underlying k-space information was acquired, are possible due to the use of a non-Cartesian radial phyllotaxis trajectory. However, since this involves segmented acquisition into a series of interleaves, the spiral phyllotaxis is less uniform in the kz direction, resulting in a higher sampling density near the equatorial plane [23], [33]. When interleaves are taken in a sequential order, the sampling becomes non-uniform, potentially leading to ringing or streaking artifacts [34]. This characteristic may bias our retrospective analyses by an increasing k-space non-uniformity when undersampling datasets, especially for a low number of radial interleaves in the simulated 1-min acquisitions. The difference between prospectively and retrospectively shortened scans was assessed by using a numerical phantom simulation, provided in the Supplementary Information (Fig. S2). This effect was mitigated by computing the density compensation using Voronoi diagrams to avoid the assumption of uniformly distributed data for each sector of sphere [24], [25]. Due to these differences and their impact on our computed metrics, we acquired data for a shortened duration of 3 min. This non-uniformity in time prevents the ability to prospectively stop the scan if a good enough image quality is achieved, which could be highly desirable, especially for pediatric and real-time MRI. In future studies, the spiral phyllotaxis trajectory could be modified to have better k-space uniformity over time or other trajectories for free-running MRI should be considered with this scope in mind.

Due to the purely data-driven nature of the SIMBA reconstruction, we can significantly reduce scan times without particular considerations for the heart or respiratory rate of each patient. However, because we take SI readouts as input, the performance of the motion correction is highly impacted by the quality of this information and its sampling frequency. A bright signal from the heart blood pool is crucial for tracking the cardiac motion as a volumetric curve over time. Thanks to ferumoxytol, this is guaranteed for each scan [22], but the presence of other bright signal sources in the field of view (e.g. from the chest wall, arms, spine) may affect the success of motion state clustering. Other MRI-independent physiological signals, such as the pilot tone [35], could be considered for continuous and decoupled motion monitoring during the acquisition. Moreover, the fact that SIMBA is a data-driven technique opens up the possibility to explore the same exact approach for angiography in other organs, such as the liver, pelvis, or lower extremities.

## 6 Conclusion

This study supports the hypothesis that SIMBA and XD-MC-SIMBA can significantly abbreviate the scan time of ferumoxytol-enhanced free-running acquisitions with minimal or no loss of image quality. Ferumoxytol allows to perform MR angiography exams in very short scan times, potentially significantly impacting the evaluation of CHD, especially in pediatric patients.

## Funding

This work has been funded in part by 10.13039/501100001711Swiss National Science Foundation (SNSF) Grants No. 320030_173129, 320030B_201292, PZ00P3_202140, and 310030_215604.

## Author contributions

**Salim Si-mohamed:** Writing – review & editing, Data curation. **Estelle Tenisch:** Writing – review & editing, Resources, Data curation. **Tobias Rutz:** Writing – review & editing, Resources, Data curation. **Xavier Sieber:** Resources. **Ludovica Romanin:** Writing – original draft, Software, Methodology, Investigation, Formal analysis, Data curation, Conceptualization. **Davide Piccini:** Writing – review & editing, Supervision. **Matthias Stuber:** Writing – review & editing, Supervision, Resources, Project administration, Funding acquisition. **Jérôme Yerly:** Writing – review & editing, Software. **Bastien Milani:** Writing – review & editing, Software, Methodology. **Prsa Milan:** Writing – review & editing, Resources, Data curation. **Christopher W. Roy:** Writing – review & editing, Methodology, Conceptualization.

## Declaration of competing interests

L.R.’s PhD studies are financially supported by Siemens Healthcare (Erlangen, Germany). At the time of the study, D.P. was an employee of Siemens Healthineers International AG (Lausanne, Switzerland) and is now an employee of Siemens Healthcare Srl (Italy). M.S. receives non-monetary research support from Siemens Healthcare (Erlangen, Germany). M.S. is a senior advisor of JCMR. The other authors have no competing financial interests or personal relationships.

## Data Availability

Data are available upon reasonable request for researchers who meet the criteria for access to confidential data.
