# Supplementary material for: Exploring the limits of scan time reduction for ferumoxytol-enhanced whole-heart angiography in congenital heart disease patients
Source: J Cardiovasc Magn Reson. 2025 Feb 5;27(1):101854. doi: 10.1016/j.jocmr.2025.101854 (PMC11889962; doi:10.1016/j.jocmr.2025.101854)
Supplement: Supplementary file 1 — Supplementary material [file mmc1.docx]

# Supplementary Information

## Figure S1. Example cases for different ranges of diagnostic quality scores

Case for which the assigned image quality scores were below good quality for all undersampling levels.

The presence of a flow-dephasing artefact (red arrow) prevents the visualization of the vessel wall, making these images of poor diagnostic quality.

##
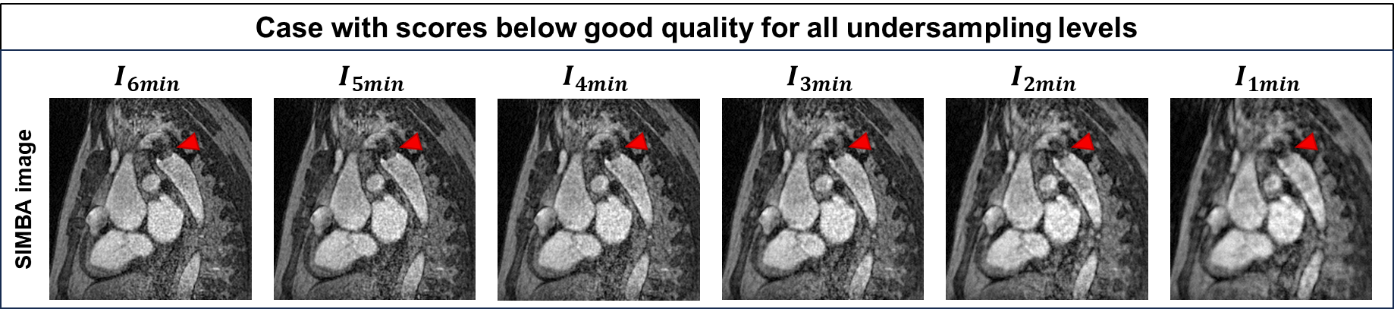


## Figure S2. Analysis of the trajectory uniformity with a numerical phantom simulation

Free-running 3D radial data was simulated using a numerical phantom simulation based on the Magnetic Resonance extended Cardiac-Torso (MRXCAT) [1] and extended Cardiac-Torso Cardiovascular Magnetic Resonance (XCMR) approaches [2,3]. The mask from the SIMBA clustering of one patient was applied to static numerical phantom data, which was then reconstructed using a 3D gridded reconstruction for non-Cartesian data. For the retrospective study, we undersampled a 6-minute acquisition into 5, 4, 3, 2, 1-minute datasets the same way it was done in our study, while for the prospective study we simulated data with different number of readouts to match the same simulated scan times.

The figure shows the readouts in the SIMBA cluster in k-space, with kx and ky directions in the x and y axis respectively. Next to it, the corresponding reconstructed image.

We can observe how the distribution of the data is different between retrospectively and prospectively undersampled datasets, and how the higher the undersampling factor (or the lower the acquisition time) the more we observe big jumps in k-space, which has a higher impact on the final image quality. This is because of non-uniform sampling in the kz direction when using spiral phyllotaxis patterns.


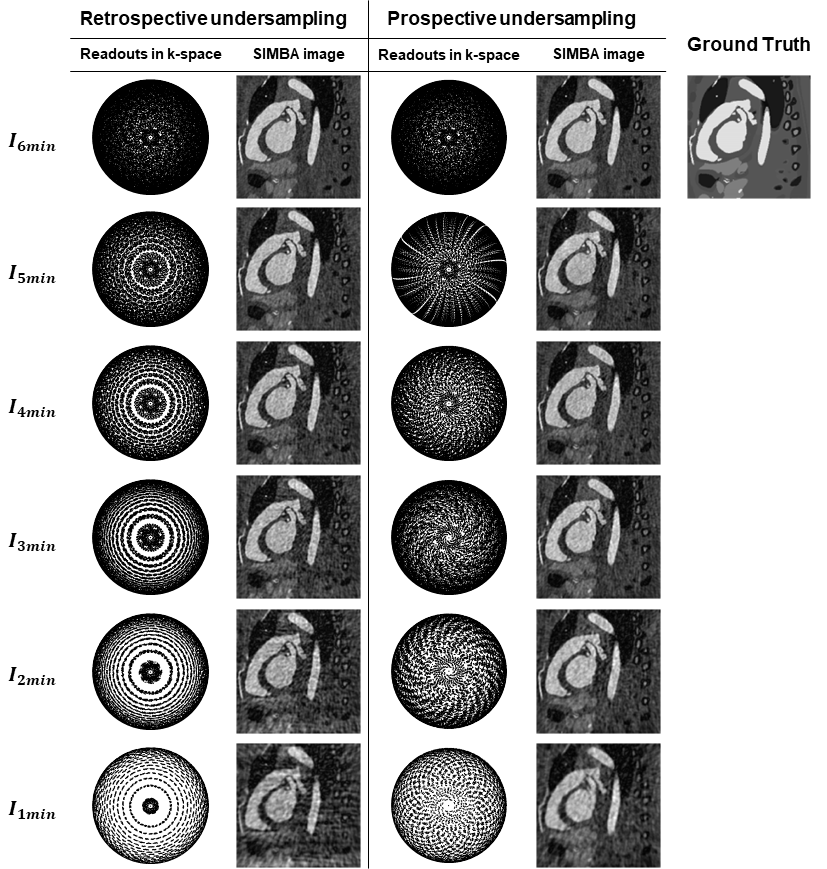


## References

1. Wissmann L, Santelli C, Segars WP, Kozerke S. MRXCAT: Realistic numerical phantoms for cardiovascular magnetic resonance. Journal of Cardiovascular Magnetic Resonance. 2014;16:63.

2. Roy CW, Marini D, Segars WP, Seed M, Macgowan CK. Fetal XCMR: a numerical phantom for fetal cardiovascular magnetic resonance imaging. Journal of Cardiovascular Magnetic Resonance. 2019;21:29.

3. Roy CW, Heerfordt J, Piccini D, Rossi G, Pavon AG, Schwitter J, et al. Motion compensated whole-heart coronary cardiovascular magnetic resonance angiography using focused navigation (fNAV). J Cardiovasc Magn Reson. 2021;23:33.

## 
